# A comparison of FLT to FDG PET/CT in the early assessment of chemotherapy response in stages IB–IIIA resectable NSCLC

**DOI:** 10.1186/s13550-017-0258-3

**Published:** 2017-01-19

**Authors:** John P. Crandall, Abdel K. Tahari, Rosalyn A. Juergens, Julie R. Brahmer, Charles M. Rudin, Giuseppe Esposito, Deepa S. Subramaniam, Michael V. Knopp, Nathan C. Hall, Prateek Gajwani, Jeffrey P. Leal, Martin A. Lodge, Joo H. O., Edward W. Gabrielson, Lalitha K. Shankar, Richard L. Wahl

**Affiliations:** 1 Mallinckrodt Institute of Radiology, Washington University School of Medicine, 510 S. Kingshighway Blvd, Campus Box 8131, St. Louis, MO 63110 USA; 2 Medical Imaging Department, King Fahad Specialist Hospital, P.O. Box 15215, Dammam, 31444-34 Saudi Arabia; 3 Juravinski Cancer Centre, McMaster University, 699 Concession Street, Fourth, Hamilton, Ontario L8V 5C2 Canada; 4 Sidney Kimmel Comprehensive Cancer Center, Johns Hopkins University School of Medicine, 1650 Orleans St, CRB I Room G-94, Baltimore, MD 21287 USA; 5 Thoracic Oncology Service, Division of Solid Tumor Oncology, Department of Medicine, Memorial Sloan Kettering Cancer Center and Weill Cornell Medical College, 633 3rd Ave, New York, NY 10017 USA; 6 Department of Radiology, Georgetown University Hospital, 3800 Reservoir Road NW CCC Bldg., Washington, DC 20007 USA; 7 Department of Medicine, Georgetown University Hospital, 3800 Reservoir Road NW LCCC Bldg., Second Floor Pod B, Washington, DC 20007 USA; 8 Department of Radiology, The Ohio State University, Wexner Medical Center, 395 W. 12th Ave., Room 430, Columbus, OH 43210 USA; 9 Department of Radiology, Perelman School of Medicine, University of Pennsylvania, 116 Donner, HUP 3400 Spruce Street, Philadelphia, PA 19104 USA; 10 The Johns Hopkins Wilmer Eye Institute, Johns Hopkins University School of Medicine, 600 N. Wolfe Street, Baltimore, MD 21287 USA; 11 The Russell H. Morgan Department of Radiology, Johns Hopkins University School of Medicine, Nelson B1-160, 600 N. Wolfe St., Baltimore, MD 21287 USA; 12 Department of Nuclear Medicine, Seoul St. Mary’s Hospital, Catholic Medical Center, Seocho-gu, Banpo-daero 222, Seoul, 06591 Korea; 13 Department of Pathology, Johns Hopkins University School of Medicine, 1550 Orleans Street, 304 CRB II, Baltimore, MD 21287 USA; 14 National Cancer Institute, 6130 Executive Boulevard, MSC 7412, Bethesda, MD 20892 USA

**Keywords:** FDG PET/CT, FLT PET/CT, Non-small cell lung cancer, Early treatment response monitoring

## Abstract

**Background:**

The aim of this study was to compare the percentage change in ^18^F-fluorothymidine (FLT) standard uptake value (SUV) between baseline and after one cycle of chemotherapy in patients categorized by RECIST 1.1 computed tomography (CT) as responders or non-responders after two cycles of therapy. Change in ^18^F-fluorodeoxyglucose (FDG) uptake was also compared between these time points.

Nine patients with newly diagnosed, operable, non-small cell lung cancer (NSCLC) were imaged with FDG positron emission tomography/CT (PET), FLT PET/CT, and CT at baseline, following one cycle of neoadjuvant therapy (75 mg/m^2^ docetaxel + 75 mg/m^2^ cisplatin), and again after the second cycle of therapy. All patients had a biopsy prior to enrollment and underwent surgical resection within 4 weeks of post-cycle 2 imaging.

**Results:**

Between baseline and post-cycle 1, non-responders had mean SULmax (maximum standard uptake value adjusted for lean body mass) increases of 7.0 and 3.4% for FDG and FLT, respectively. Responders had mean decreases of 44.8 and 32.0% in FDG and FLT SULmax, respectively, between baseline and post-cycle 1 imaging. On post-cycle 1 imaging, primary tumor FDG SUL values were significantly lower in responders than in non-responders (*P* = 0.016). Primary tumor FLT SUL values did not differ significantly between these groups. Using the change from baseline to post-cycle 1, receiver-operating characteristic (ROC) analysis showed an area under the curve (AUC) of 0.94 for FDG and 0.78 for FLT in predicting anatomic tumor response after the second cycle of therapy.

**Conclusions:**

Fractional decrease in FDG SULmax from baseline to post-cycle 1 imaging was significantly different between anatomic responders and non-responders, while percentage changes in FLT SULmax were not significantly different between these groups over the same period of time.

## Background

Lung cancer is the most common cause of cancer-related mortality worldwide [1]. The three modalities most commonly used to treat cancer have been surgery, radiation therapy, and systemic chemo (and now, immune) therapy. Surgical resection likely offers the best chance for cure, especially in patients with stage I or II disease [2, 3]. Giving chemotherapy before surgery, “neoadjuvant chemotherapy,” has potential benefits including reduction of tumor size, eradication of micrometastases including in nodes, and tumor downstaging, which may allow for a more complete and potentially curative resection. Currently, The NCCN (National Comprehensive Cancer Network) Guidelines recommend induction (neoadjuvant) chemotherapy for patients with stage II or IIIA disease, though the appropriate role for neoadjuvant chemotherapy is still evolving [2]. SWOG 9900 was a phase III trial of surgery alone or surgery plus induction paclitaxel/carboplatin chemotherapy in early stage non-small cell lung cancer (NSCLC) [4]. In this study, 354 patients with stages IB–IIIA NSCLC were randomized to receive induction chemotherapy versus no induction chemotherapy. The study closed prematurely and was likely underpowered, but in the chemotherapy arm, a trend toward improved progression-free survival and overall survival was shown. A 2014 study by the NSCLC Meta-Analysis Collaborative Group analyzed 15 randomized controlled trials of patients with stages IB–IIIA NSCLC and concluded that preoperative chemotherapy significantly improves overall survival and recurrence-free survival in defined populations [5].

In cancer therapy, having a mechanism through which treatment efficacy can be monitored, ideally soon after initiation of treatment would be valuable, especially in diseases such as lung cancer where the minority of patients have an objective response. Early identification of patients who are unlikely to benefit from induction therapy is important as they could be saved from unnecessary side effects of ineffective treatment and potentially avoid further delay of surgical resection. Various imaging modalities have been used for this purpose in the clinical setting. One formalized system of assessing anatomic tumor response is the Response Evaluation Criteria in Solid Tumors (RECIST), which is based on serial measurements using standard imaging techniques such as computed tomography (CT) [6]. This method relies on changes in tumor size, which is likely a crude surrogate for alterations in tumor proliferation or cell death, which may occur much sooner than tumor shrinkage. Thus, evaluation of methods that can be applied to assess response at earlier time points during treatment is important.

One attempt to ascertain the biology of lesions seen on CT imaging has been with positron emission tomography (PET). PET with ^18^F-fluorodeoxyglucose (FDG) is standard of care for the initial staging of patients with NSCLC. Sequential FDG PET imaging has also been investigated as a metric of response to treatment [7–9]. Increased FDG uptake in tumors is generally correlated positively with the total tumor cell mass, and declines in FDG uptake with treatment are typically associated with response to therapy [10, 11]. Using (FDG) PET imaging to assess response will, in many cancers, typically demonstrate rapid reduction in tumor (^18^F-FDG) signal with effective therapy, a decline not uncommonly antedating decrease in tumor size [12, 13]. FDG PET carries with it challenges in assessing response, notably uptake of the radiotracer into non-malignant inflammatory cells, which can confound assessments of tumor response. In addition, “flare” reactions and “stunning” of FDG activity levels by treatment have been described, which make it less than perfect in some instances as a general early metric of tumor response to treatment.

Another radiotracer that has been investigated for use with PET is ^18^F-fluorothymidine (FLT). FLT is an F-18-labeled pyrimidine analog, that is a substrate for thymidine kinase. Once transported and phosphorylated, the molecule is trapped within the cell but not able to be incorporated into DNA [14]. Thymidine kinase concentrations have been shown to be elevated tenfold or more in cells with active DNA synthesis, such as in malignant cells [15]. Thus, FLT uptake is postulated to be a marker of active DNA synthesis in vivo.

18F-FLT was first used in humans by Shields and colleagues in 1998 [16]. Since that time, studies have been performed to assess its use as a marker of tumor proliferation in vivo [17, 18]. Buck and colleagues in 2003 conducted one of the initial studies which assessed the correlation between FLT uptake and lung tumor proliferation in humans [17]. Tumor proliferation was quantified using a Ki-67-specific monoclonal antibody in tumor specimens. In this study, which included 26 patients, increased FLT uptake correlated well with Ki-67 staining in malignant primary lung tumors. Further studies have been conducted in pre-clinical models assessing the utility of FLT PET in assessing tumor response to chemotherapy. Leyton and colleagues assessed the response of radiation-induced fibrosarcomas to cisplatin in mice [19]. Using immunohistochemistry for proliferating cell nuclear antigen, decreased FLT uptake was correlated with decreased cell proliferation seen pathologically prior to evidence of change in tumor size measured grossly with calipers. Kostakoglu et al. assessed the ability of changes in the FLT PET/CT signals performed before and following one cycle of neoadjuvant chemotherapy to predict pathological response assessed after a second cycle of neoadjuvant chemotherapy in breast cancer patients [20]. Results from that study, which included patients undergoing various neoadjuvant regimens, showed FLT PET/CT imaging after one cycle of neoadjuvant chemotherapy weakly predicted pathological complete response.

The aim of this study was to evaluate and compare FLT and FDG imaging, performed at baseline and after one cycle of neoadjuvant chemotherapy in newly diagnosed non-small cell lung cancer patients categorized as responders or non-responders based on RECIST 1.1 using CT measurements after the second cycle of therapy.

## Methods

This study of FDG and FLT PET/CT imaging in patients with NSCLC undergoing neoadjuvant chemotherapy was a phase II, open-label multicenter (three sites accrued patients: Johns Hopkins University, Ohio State University, and Georgetown University) trial and was performed in accordance with the Johns Hopkins Medicine Institutional Review Board under a Food and Drug Administration investigational new drug application (IND 71260). Written, informed consent was obtained from all patients and the trial was registered on http://www.clinicaltrials.gov with the identifier NCT00963807. Pertinent inclusion criteria for this study included newly diagnosed patients with stages IB–IIIA NSCLC who were eligible for surgical resection, had measureable disease per RECIST 1.1, and were candidates for platinum-based chemotherapy regimens. Pertinent exclusion criteria included prior history of any other malignancy within the last 3 years other than non-melanoma skin cancer and in situ carcinoma of the cervix, a history of prior radiation therapy or systemic chemotherapy for lung cancer, elevated bilirubin, peripheral neuropathies greater than grade 1, and patients with baseline hearing loss.

Sample size for this prospective study was based on several parameters. Using the data from Fossella et al., we postulated a radiographic combined partial and complete response rate of 30% for the combination of docetaxel and cisplatin [21]. This would, on average, place three out of every ten patients in the response category and seven out of every ten patients in the non-response category. A clinically significant difference between non-responding and responding patients in the fractional difference in FLT uptake between the baseline scan and the scan obtained after the first cycle of treatment was determined to be at least one standard deviation. Based on a two-sided type I error allowance of 5% and a power of 90% using a one standard deviation change as the difference hypothesized to be detected, a total sample size of 55 (16 responders and 39 non-responders) was calculated. However, this target was not reached because federal ARRA (American Recovery and Reinvestment Act) funding for the study was time limited and accrual was less rapid than expected.

**Fig. 1:**
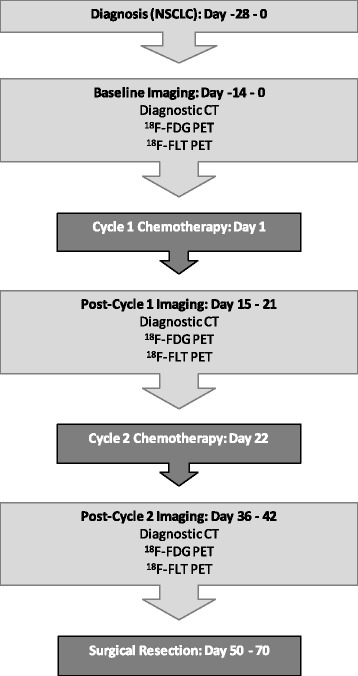
Patient flow diagram

The patient flow diagram is summarized in Fig. 1. Eligible patients received a standard-of-care, neoadjuvant regimen of concurrent docetaxel plus cisplatin. Both agents were administered once every 3 weeks for two total cycles, each at a dose of 75 mg/m^2^. Surgical resection (of the tumor) was performed within 8–10 weeks of the start of chemotherapy.

Patients underwent whole body PET/CT imaging (FLT and FDG), as well as non-contrast thoracic CT at baseline, 2–3 weeks following cycle 1 chemotherapy, and 2–3 weeks following cycle 2 chemotherapy. FDG PET/CT imaging and patient preparation was performed as outlined by Shankar et al. [22]. FLT PET/CT and FDG PET/CT imaging was completed within 7 days and at least 24 h apart. All nuclear imaging was performed on a full-ring PET/CT scanner with the capability of quantitative SUV determination. To help ensure consistency, each site was asked to acquire and reconstruct a phantom study for central site review, as well as submit a representative patient study. The phantom and patient study were reviewed for a variety of quantitative features (e.g., uniformity, Max & Mean SUV values, etc.) before the site was qualified for participation in the trial. All sites used a uniform imaging protocol which included an FLT dose and uptake time of 370 Mbq (+/− 20%) and 80 min (+/− 10 min), respectively. On a separate day, FDG was administered per each institution’s standard and uptake time was 60 min (+/− 10 min). The median administered dose of FDG was 10.8 mCi with a range of 8.0–14.8 mCi. Thoracic CT with full inspiration breath-hold was obtained at each imaging time point on the PET/CT scanner immediately before either the FLT PET/CT scan or the FDG PET/CT scan.

### Image analysis

A single board-certified nuclear medicine physician, blinded to clinical outcomes and pathology results, used a clinical imaging workstation (Mirada XD3, Mirada Medical, Denver, CO) to determine SUV data for the FDG and FLT scans. A large volume of interest (VOI) to include the entire primary tumor was manually drawn, and the maximum voxel value was recorded within the VOI. The CT tumor longest dimensions were determined separately by a single, board-certified imaging specialist.

### Immunohistochemistry

A tumor biopsy was obtained at baseline before neoadjuvant chemotherapy and at the time of surgical resection. Slides were stained for Ki-67, caspase-3, Glut-1, ERCC1, and CHFR using commercially available monoclonal antibodies. All immunohistochemistry results were obtained in a CLIA (Clinical Laboratory Improvement Amendment)-certified laboratory and quantified at the core site by the study pathologist, without reference to the PET or CT scan data.

### Statistical considerations

The primary endpoint was percentage change in FLT SUVmax from baseline to cycle 1 of chemotherapy in patients characterized as responders or non-responders based on RECIST measurements obtained following cycle 2 of chemotherapy. Analysis of variance was used to test the differences in FDG and FLT uptake between responders and non-responders at various time points. In cases of dissimilar variance between groups, a log-transformation was performed prior to the *t* test. Regression analysis was used to estimate the relationships between baseline FLT SULpeak and baseline Ki-67 index, as well as between FLT and FDG SUVmax/SULmax at cycle 1 and change in tumor size measured on CT following cycle 2. The three imaging modalities (FLT PET/CT, FDG PET/CT, and diagnostic CT) were compared using receiver-operating characteristic (ROC) curve analysis. In all analyses, a *P* value of less than 0.05 was considered statistically significant. Descriptive statistics were calculated using Microsoft Excel, and further analyses were performed with Prism4.0 (Graphpad Software).

## Results

Twenty-six patients were prospectively enrolled between October 2009 and March 2012. Following informed consent and prior to baseline imaging, 9 patients were withdrawn from the study due to disease progression, 2 for baseline hearing loss, and 1 for elevated bilirubin. Two patients chose not to participate due to the number of scans required, and 1 patient chose not to participate because he or she did not wish to undergo chemotherapy. The remaining 11 patients underwent baseline imaging including FLT PET/CT, FDG PET/CT, and diagnostic CT. One patient was excluded following baseline imaging due to disease progression, and another was removed following cycle 1 imaging due to toxicity to the chemotherapy regimen.

**Fig. 2:**
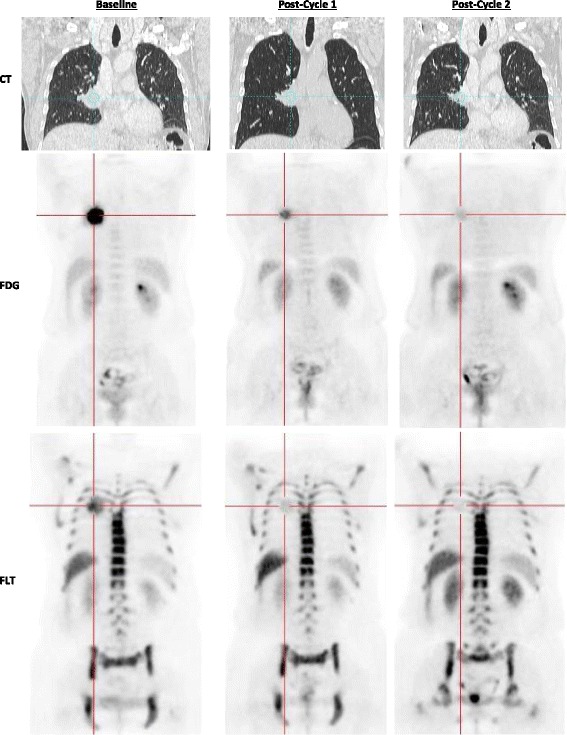
Representative images of a patient (patient 2) classified as a “responder”. CT, FDG, and FLT images are shown at each indicated time point. The same lesion is targeted in each image

Characteristics of the remaining 9 patients are summarized in Table 1. For these 9 patients, the median between FDG and FLT was 3 days (range 1–17 days), and between FLT and commencement of chemotherapy was 3 days (range 1–8 days). All 9 patients who underwent baseline imaging and proceeded with initiation of chemotherapy underwent post-cycle 1 imaging. One patient did not undergo post-cycle 2 FDG imaging due to a complication related to the chemotherapy regimen, though he did complete FLT and diagnostic CT imaging. All scans for a given patient and tracer were obtained on the same scanner. A series of representative patient images are shown in Fig. 2.

**Table 1:** Patient characteristics, clinical TNM stage, and treatment response as determined by RECIST following 2 cycles of therapy

Patient no.	Age	Gender	BMI	Stage	T	N	M	Histology	Cycle 2 RECIST response
1	60	M	27.4	IIB	2b	1	0	Squamous	Non-responder (SD)
2	59	F	32.3	IIIA	3	1	0	Squamous	Responder (PR)
3	77	M	27.8	IIA	1b	1	0	Adenocarcinoma	Non-responder (SD)
4	50	M	25.1	IIIA	3	2	0	Squamous	Non-responder (SD)
5	55	M	34.6	IIB	2b	1	0	Squamous	Non-responder (SD)
6	33	M	35.9	IIA	2b	0	0	Adenocarcinoma	Responder (PR)
7	60	M	29.1	IIIA	1b	2	0	Adenocarcinoma	Non-responder (SD)
8	45	F	17.3	IIB	3	0	0	Adenocarcinoma	Non-responder (SD)
9	69	F	17.6	IIB	3	0	0	Squamous	Responder (PR)

Primary tumor uptake of FDG was significantly higher than FLT at all time points (*P* < 0.01). At baseline, average SUVmax/SULmax was 13.0 ± 5.8/8.8 ± 3.4 for FDG and 5.6 ± 2.0/3.8 ± 1.2 for FLT. On post-cycle 1 imaging, average SUVmax/SULmax was 10.5 ± 5.1/7.3 ± 3.4 for FDG and 4.8 ± 2.5/3.4 ± 1.8 for FLT. On post-cycle 2 imaging, average SUVmax/SULmax was 7.4 ± 3.6/5.1 ± 2.4 for FDG and 4.4 ± 2.3/4.0 ± 4.1 for FLT. Based on RECIST 1.1 criteria using CT measurements following cycle 2 of chemotherapy, 3 patients were classified as responders (0 complete responders; 3 partial responders) and 6 patients as non-responders (6 stable disease; 0 progressive disease). Uptake values and baseline tumor size measurements, as well as percentage change in tumor size at both follow-up time points, can be found in Table 2.

**Table 2:** FDG SULmax and FLT SULmax values at all time points for each patient

Patient number	Baseline			Post-cycle 1			Post-cycle 2		
	FDG	FLT	CT	FDG	FLT	CT (%)	FDG	FLT	CT (%)
1	9.3	3.4	6.5	8.8	6.8	3.1	4.1	2.2	−7.7
2*	12.7	5.2	7.4	3.4	1.7	−45.9	1.5	1.0	−56.8
3	3.2	2.3	2.4	4.0	1.0	4.2	3.3	1.1	−8.3
4	8.3	4.5	8.6	6.7	2.9	44.2	–	14.0	−16.3
5	14.0	4.6	5.4	14.6	4.4	31.5	7.4	3.9	−29.6
6*	10.9	4.9	5.9	8.9	4.2	−25.4	8.6	4.4	−52.5
7	5.5	2.7	2.1	7.7	2.9	0.0	5.4	2.5	−14.3
8	7.1	4.2	11.5	6.9	4.6	−23.5	6.7	5.3	−27.8
9*	8.4	2.1	8.4	4.8	1.8	−21.4	3.9	1.4	−44.0

Post-cycle 1 and post-cycle 2 CT values are shown as percentage changes from baseline. Responders are denoted by an asterisk after the patient number

**Fig. 3:**
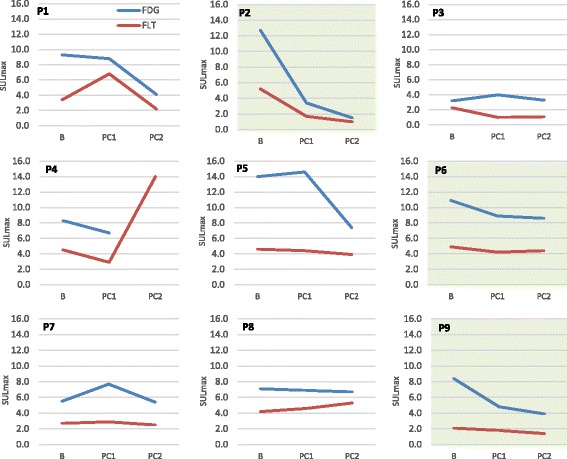
Line graphs showing FDG SULmax and FLT SULmax at baseline (B), post-cycle 1 (PC1), and post-cycle 2 (PC2). In responding patients (*highlighted in green*), FDG SULmax decreased significantly more from baseline to post-cycle 1 than in non-responding patients (*P* = 0.017). FLT SULmax did not differ significantly between responders and non-responders (*P* = 0.336) from baseline to post-cycle 1

Change in FDG SULmax from baseline to post-cycle 1 imaging differed significantly between responders and non-responders (Fig. 3). Responders had a mean primary tumor FDG SULmax decrease of 44.8 ± 27.5% and non-responders had an increase of 7.0 ± 21.7% (*P* = 0.017). Change in post-cycle 1 FDG SUVmax and SULpeak (not shown) also differed significantly between these groups (*P* = 0.016 and *P* = 0.026, respectively). Percentage change in tumor size as measured on CT from baseline to post-cycle 1 imaging differed significantly between responders and non-responders (*P* = 0.016). Responders had a change in FLT SULmax from baseline to post-cycle 1 imaging that was not significantly different from non-responders. Responders had a mean primary tumor FLT SULmax decrease of 32.0 ± 30.6%, and non-responders had an increase of 3.4 ± 54.0% (*P* = 0.336). Change in FLT SULpeak was also not significantly different between responders and non-responders. Change in FDG SULmax differed depending on tumor histology. The mean change in primary tumor FDG SULmax from baseline to post-cycle 2 was −61.2 ± 18.4% for squamous cell carcinomas and −6.4 ± 10.5% for adenocarcinomas (*P* = 0.002). Baseline FDG and FLT uptake did not differ significantly between responders and non-responders. Percentage change in FDG and FLT SULmax from baseline to post-cycle 2 did not differ significantly between responders and non-responders, which may be due to the small sample size and that FDG and FLT uptake was substantially lower after cycle 2 (median FDG and FLT SULmax dropped by 40% from baseline to post-cycle 2 imaging) so a significant difference among these low SUVs was not easily visualized, and supporting concepts that early imaging of response, post-cycle 1, for example, may be most informative [23].

**Fig. 4:**
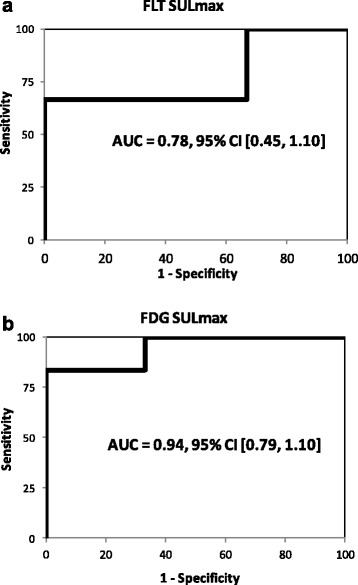
ROC curves with associated AUC values of 0.78 and 0.94 for FLT and FDG, respectively. ROC analysis showed FDG PET (**b**) after once cycle of therapy was a significant predictor of response as determined by CT after two cycles (*P* = 0.039). FLT (**a**) was not significantly predictive (*P* = 0.197)

ROC analysis (Fig. 4) indicated FLT SULmax to be no better than chance, in this small patient population, at determining responders versus non-responders following one cycle of treatment (AUC = 0.78, *P* = 0.197). Percentage change in FDG uptake following one cycle of chemotherapy was a significant predictor of post-cycle 2 CT results in an AUC of 0.94 (*P* = 0.039). The ROC analysis of FDG SULmax data indicated a drop of 31.1% between baseline and post-cycle 1 of treatment to be the ideal cutoff between responders and non-responders within this population.

Changes in fractional tumor viability and proliferative fraction were assessed by comparing differences in caspase-3 and Ki-67, respectively, between the baseline biopsy and the surgical resection performed following the second cycle of chemotherapy. This analysis showed no substantial correlation between caspase-3 and FDG or FLT SUV data. Baseline Ki-67mean index was found to be significantly correlated with baseline FLT SULpeak-total (*r* = −0.78, *P* = 0.039). This correlation differed between squamous cell carcinoma and adenocarcinoma cases. Squamous cell carcinoma cases showed a significant correlation between baseline Ki-67 and baseline FLT SULpeak-total (*r* = −0.95, *P* = 0.013), while no correlation between baseline Ki-67 and baseline FLT SULpeak-total was found for adenocarcinoma cases. Change in Ki-67 from baseline biopsy to surgical resection was not correlated with change in FDG or FLT SUV between baseline and post-cycle 2 imaging. Methylation status of the CHFR gene could not be correlated with any variable because all samples were CHFR negative.

## Discussion

In the present prospective study, we investigated the ability of FLT and FDG PET/CT to assess early response in NSCLC patients treated with a neoadjuvant, platinum-based chemotherapy regimen. Platinum doublet chemotherapy was first defined as beneficial in lung cancer in 1995 through a meta-analysis published in the *British Medical Journal* [24]. Shortly thereafter, clinicians sought surrogate methods to predict survival outcome. One of the earliest studies published identified that lack of objective response with anatomic imaging predicted for poor survival outcomes [25]. Clearly, this measure was crude. In our study, changes in SUV data between baseline and following one cycle of therapy were compared with anatomic CT data taken at baseline and after two cycles of therapy. Despite our relatively small study group, change in FDG SUV after one cycle of therapy showed significant association with response as determined by CT following the second cycle of therapy. In other studies of NSCLC patients, early FDG PET has been shown useful in predicting tumor response [8, 9, 12]. Weber et al. evaluated 57 stage IIIB/IV NSCLC patients with FDG PET before and after the first cycle of a platinum-based chemotherapy regimen. Metabolic response on PET was significantly correlated with overall response as determined by RECIST using CT results after two cycles of therapy [12]. Twenty-three patients in a phase II study on response to neoadjuvant erlotinib underwent FDG PET at baseline and within 7 days after the first dose of chemotherapy, followed by surgical resection. In patients classified as “metabolic responders” (an SUVmax decrease by more than 25%), the median percentage necrosis was 70%, while the median percentage necrosis in metabolic non-responders was 40%, with a *P* value of 0.09 [9]. More recently, in a retrospective cohort study, Han and colleagues have correlated overall survival with both baseline metabolic uptake as well as change in SUVmax after chemotherapy in advanced NSCLC. This supports our premise that PET could be used in both a prognostic and predictive fashion in NSCLC [26].

At post-cycle 1 imaging, FLT PET was not a substantial predictor of response in this small trial. Studies on response evaluation using FLT have produced conflicting results. Sohn and colleagues assessed percent change in FLT SUVmax between baseline and 7 days after the start of gefitinib therapy in 28 patients with adenocarcinoma of the lung. Chest CT after 6 weeks of treatment was used to determine response status. Responders were found to have a significantly different change in SUVmax than non-responders (−36.0 ± 15.4% versus 10.1 ± 19.5%, respectively; *P* < 0.001) [27]. Another study by Bhoil and colleagues looked at 15 NSCLC patients who underwent imaging at baseline and after 3 weeks of EGFR kinase inhibitor treatment. In this cohort, change in FDG SULpeak from baseline to 3 weeks post-treatment was significantly better than change in FLT SULpeak at predicting overall survival and progression-free survival [28]. PET characteristics may differ when assessing cytotoxic chemotherapeutics in contrast to targeted therapies such as EGFR inhibitors depending on the mutational status of the tumor. Our small series is consistent with that of Weber et al. who also evaluated response to a platinum doublet.

Baseline proliferative fraction, assessed using Ki-67mean index, was shown to be inversely correlated with baseline FLT SULpeak-total. Other clinical studies have shown conflicting results on the relationship between Ki-67 expression and FLT uptake, with some studies confirming a good correlation and others presenting negative results. One study in 10 patients with esophageal cancer reported an inverse correlation between FLT SUVmax and Ki-67max [29]. Several biological explanations have been offered for the lack of correlation between FLT and Ki-67: the absence of cell cycle-specific regulation of thymidine kinase 1 [30]; the heterogeneity of Ki-67 expression within tumor samples; variations in cellular ATP levels; Ki-67 expression being related to cellular proliferation via the salvage pathway as well as the de novo pathway, while FLT uptake is likely only related via the salvage pathway [31]. A recent meta-analysis suggested that the methodology used in studies comparing FLT uptake and Ki-67 expression may have a significant impact on the FLT/Ki-67 correlation [32]. Specifically, studies utilizing surgical tissue samples and assessing Ki-67mean and FLT SUVmax tend to produce higher correlation coefficients. Additionally, it is possible that dynamic FLT studies, as opposed to single static SUV images, could elucidate a stronger positive correlation between proliferation and SUV [33]. It is also possible the mixture of both adenocarcinomas and squamous cell carcinomas in our small population could mask a positive correlation.

Correlations between percentage changes in viable tumor are only modest for both tracers. However, the metric used does not take into consideration total tumor size and percent viability multiplied by tumor volume may likely be a more relevant parameter. Similarly, since none of our patients had abnormal methylation status at baseline, no conclusions can be drawn regarding promoter methylation and response.

A key limitation of the current study was the small population size. We were unable to reach our targeted accrual goal of 55 patients because the study was time limited due to ARRA funding. Patients often chose not to participate due to the conventional chemotherapy offered in the study given the alternative options of chemoradiation and other targeted therapies. The ambitious study design, with six PET/CT scans, was difficult for patients to agree to. Given these limitations, the study was not powered to determine if FLT or FDG were different from one another in assessing treatment response. However, it is interesting that the limited data suggest FDG to be more accurate than FLT in the task of predicting response as determined by RECIST at cycle 2 and that the decline in FDG SUL was larger, as a percentage, than the decline in SUL for FLT in the responding patients.

In our study, FLT baseline uptake in NSCLC is significantly lower compared with FDG uptake. Fractional decrease in FDG SULmax from baseline to post-cycle 1 imaging is significantly larger in responders than in non-responders, while change in FLT SULmax is not significantly different between these groups over the same period of time. ROC analysis indicates FDG PET after one cycle of therapy is a better predictor of outcome than FLT PET. Our data do not suggest a compelling advantage for FLT versus FDG in early assessment of chemotherapy response in NSCLC.

## Conclusions

FLT PET has been postulated to predict proliferation status, and previous studies have indicated that FLT PET imaging may provide beneficial information early on that could help guide treatment decisions. In contrast to these studies, the results in this small study suggest FLT PET imaging offers no significant advantage over FDG PET imaging in early chemotherapy response prediction in lung cancer. In addition, this study highlights the predictive ability of early FDG PET imaging, which is consistent with other studies in similar patient populations. Further studies with larger sample sizes would be informative to strengthen our conclusion.

## Abbreviations

ARRA: American Recovery and Reinvestment Act
CLIA: Clinical Laboratory Improvement Amendment
CT: Computed tomography
FDG: ^18^F-fluorodeoxyglucose
FLT: ^18^F-fluoro-l-thymidine
MRI: Magnetic resonance imaging
NCCN: National Comprehensive Cancer Network
NSCLC: Non-small cell lung cancer
PET: Positron emission tomography
RECIST: Response Evaluation Criteria in Solid Tumors
ROC: Receiver operator characteristic
SUL: SUV normalized by lean body mass
SULpeak: Average SUV computed within a volume of interest
SUV: Standard uptake value
SUVmax: Maximum standardized uptake value
VOI: Volume of interest
